# Hybrid Metal-Dielectric-Metal Sandwiches for SERS Applications

**DOI:** 10.3390/nano11123205

**Published:** 2021-11-26

**Authors:** Mikhail K. Tatmyshevskiy, Dmitry I. Yakubovsky, Olesya O. Kapitanova, Valentin R. Solovey, Andrey A. Vyshnevyy, Georgy A. Ermolaev, Yuri A. Klishin, Mikhail S. Mironov, Artem A. Voronov, Aleksey V. Arsenin, Valentyn S. Volkov, Sergey M. Novikov

**Affiliations:** 1 Center for Photonics and 2D Materials, Moscow Institute of Physics and Technology (MIPT), 9 Institutsky Lane, 141700 Dolgoprudny, Russia; dmitrii.yakubovskii@phystech.edu (D.I.Y.); olesya.kapitanova@gmail.com (O.O.K.); valentinsr@mail.ru (V.R.S.); andrey.vyshnevyy@phystech.edu (A.A.V.); ermolaev-georgy@yandex.ru (G.A.E.); klishin.yuri@mail.ru (Y.A.K.); mironov.ms@phystech.edu (M.S.M.); voronov.artem@gmail.com (A.A.V.); arsenin.av@mipt.ru (A.V.A.); vsv.mipt@gmail.com (V.S.V.); 2 Department of Chemistry, Lomonosov Moscow State University, 1-3 Leninskiye Gory, 119991 Moscow, Russia

**Keywords:** plasmonics, field enhancement, surface-enhanced Raman scattering, linear spectroscopy, ultrathin gold films, graphene, percolation threshold, gap surface plasmons

## Abstract

The development of efficient plasmonic nanostructures with controlled and reproducible surface-enhanced Raman spectroscopy (SERS) signals is an important task for the evolution of ultrasensitive sensor-related methods. One of the methods to improving the characteristics of nanostructures is the development of hybrid structures that include several types of materials. Here, we experimentally investigate ultrathin gold films (3–9 nm) near the percolation threshold on Si/Au/SiO_2_ and Si/Au/SiO_2_/graphene multilayer structures. The occurring field enhanced (FE) effects were characterized by a recording of SERS signal from Crystal Violet dye. In this geometry, the overall FE principally benefits from the combination of two mechanisms. The first one is associated with plasmon excitation in Au clusters located closest to each other. The second is due to the gap plasmons’ excitation in a thin dielectric layer between the mirror and corrugated gold layers. Experimentally obtained SERS signals from sandwiched structures fabricated with Au film of 100 nm as a reflector, dielectric SiO_2_ spacer of 50 nm and ultrathin gold atop could reach SERS enhancements of up to around seven times relative to gold films near the percolation threshold deposited on a standard glass substrate. The close contiguity of the analyte to graphene and nanostructured Au efficiently quenches the fluorescent background of the model compound. The obtained result shows that the strategy of combining ultrathin nano-island gold films near the percolation threshold with gap plasmon resonances is promising for the design of highly efficient SERS substrates for potential applications in ultrasensitive Raman detection.

## 1. Introduction

Surface-enhanced Raman spectroscopy (SERS) is an analytical tool that allows detecting chemical compounds at extremely low concentrations (down to single molecules) and provides unique spectra based on the specific vibration bonds of molecules [1,2,3]. SERS is achieved due to strong electromagnetic field enhancement (FE), which occurs due to the excitation of plasmons in resonant metal nanostructures [4,5,6,7]. Substantially, the requirements for SERS structures can be listed as follows: simplicity, reliability, and low manufacturing cost, as well as high reproducibility and uniformity over a large area. Note that highly sensitive substrates, for instance, for single-molecule level detection are not always required. In some cases, reproducible and satisfactory signal amplification is sufficient. Therefore, the requirements for the sensitivity of SERS structures can be different for each specific case. Here, we have focused on highly sensitive and reproducible SERS substrates for potential analytical applications. One of the contenders for such structures is SERS substrates based on semi-continuous percolated metal films [8,9,10]. The method of fabrication of such substrates is one of the easiest and cheapest. For metal films, the percolation threshold is the key point at which individual metal clusters start forming into connected structures. Near the percolation threshold, the clusters of metal are located close to each other and form so-called “hot spots” [11,12]. The concentration of such hot spots is at the maximum near the percolation threshold. The strongest value of FE near the percolation threshold was demonstrated in previous studies by optical near-field and two-photon luminescence microscopy [8,12,13]. As a result of strong FE, the amplification of the second harmonic generation and the increase in SERS signal were observed [9,14,15,16]. Despite all the advantages associated with the simplicity of production and high reproducibility (both in manufacturing and in signal amplification), such structures have a relatively low gain [9,15]. However, there is an opportunity to increase the efficiency of such structures. The hybrid structures, which include several types of materials, are becoming wide spread in various fields, including SERS [17,18]. One of the examples of such structures is corrugated metal nanosurfaces separated by a thin dielectric layer from a metal substrate. Such structures can support highly confined plasmon modes, known as gap surface plasmons (GSP) [19,20,21]. The resonances of the GSP modes are associated with the excitation of magnetic-dipole current configurations with strongly suppressed radiative damping. The resonances of GSP modes can easily variate, for instance, by changing the thickness of the gap or the size and shape of the upper nanostructures [20,21,22]. These plasmonic structures allow obtaining relatively large and stable effects associated with field enhancement and, therefore, can be of particular interest for use in SERS applications. However, together with SERS amplification, the signal of luminescence also can be amplified in the same manner [23]. Since luminescence is a competing phenomenon with Raman scattering and is the source of strong background signals. Thus, it is important to enhance the SERS signal and suppress the increase in luminescence.

In this investigation, we study the efficiency of hybrid SERS structures consisting of ultrathin gold films (3–9 nm) near the percolation threshold deposited over a gold mirror (100 nm) with a SiO_2_ spacer layer (50 nm) with and without graphene. We compare the SERS enhancement factor of these hybrid structures with SERS structures consisting of ultrathin Au films with the same thicknesses but coated on glass substrates. Graphene is a promising 2D material for SERS-platforms [24,25], first of all, due to the possibility of luminescence quenching [15,26,27]. Moreover, as a metal, gold was chosen as a stable metal unaffected by oxidation, unlike silver or copper. The advantages of the multilayer SERS substrates Au/SiO_2_/Au and Au/SiO_2_/graphene/Au compared to simple glass/Au substrates are discussed. Note that similar configurations were considered earlier as structures with high light absorbers, but they were not applied as SERS structures [28,29,30]. We also note the direction associated with a similarly suggested design of substrates in which the optical interference effects provide additional enhancement of the Raman intensity [31,32]. The percolation threshold of the deposited gold films is determined by various measurement methods of sheet resistance via the four-probe method and effective permittivity via spectroscopic ellipsometry analysis. The morphology of ultrathin nano-island Au films is characterized by using scanning electron and atomic force microscopy. Measurements of SERS spectra demonstrated that signal enhancement of the hybrid substrates, compared to glass/Au substrate, is on average over ~6 times higher for hybrid substrate with graphene and over ~7 times higher for hybrid substrate without graphene and with SERS enhancement factors of up to ~10^6^. The sublayer of graphene in the hybrid substrate quenches the luminescence background of CV by an average of 40%. The presented results show that a combination of GSP resonators and films near the percolation threshold can be promising for the design of efficient SERS structures for sensing applications.

## 2. Materials and Methods

### 2.1. Sample Fabrication

The ultra-thin Au films with thicknesses ranging from 3 to 9 nm with a 1 nm step were deposited onto three types of substrates. The first type was an uncovered glass substrate. Before deposition of gold films, the glass slides were washed with a piranha solution and extensively rinsed with deionized water. The second substrate was a 100 nm layer of Au deposited on silicon substrate, 3 nm Ti adhesion interlayer over Au, and 50 nm layer of SiO_2_. A thin titanium layer was introduced as it promotes adhesion followed by deposited SiO_2_ to the gold layer. The third substrate was the same as the second one, but with a monolayer of CVD-graphene on top of the SiO_2_ layer transferred from copper foil using the PMMA-mediated method [33]. The substrate surface was covered at least 90% by transferred graphene. After the transfer, the substrates with graphene have been annealed at 250 °C in a vacuum chamber 10^−6^ Torr for 1 h to clean residual PMMA and water. All thicknesses of SiO_2_ were confirmed by ellipsometry. The Au films for all substrate types were deposited in one regime by electron beam evaporation in a Nano Master NEE-4000 (NANO-MASTER Inc., Austin, TX, USA) installation at a high vacuum, with the pressure of residual gases in the chamber being no greater than 5 × 10^−6^ Torr and deposition rate of 0.5 A/s at room temperature (21 °C). Each ultrathin gold film of different thicknesses was deposited simultaneously onto three types of substrates in one cycle. The granulated target of Au was produced by Kurt J. Lesker (East Sussex, UK) with a purity of 99.999%. The thickness of Au films was controlled by the quartz oscillator mass-thickness sensor during the deposition.

### 2.2. Electrical Measurements, Scanning Electron, and Atomic Force Microscopy

The surface morphology of the deposited films was visualized by scanning electron microscope (SEM) JEOL JSM-7001F (JEOL Ltd., Tokyo, Japan). The thickness of the films, morphology, and their roughness were measured by an atomic force microscope (NT-MDT Ntegra, Moscow, Russia). Electrical measurements were performed by a 4-probe station (Jandel Engineering Ltd., Linslade, UK) with a collinear geometry of the probes and started with a current of 1 μA, gradually increasing up to 100 μA. The measurements were carried out on at least 3 different points of the sample, and average values were taken.

### 2.3. Reflection Analysis

The reflection spectra were recorded by Biolam M-1 microscope backscattering configuration, equipped with a 24 V, 100 W halogen light source, polarizers, and a fiber-coupled grating spectrometer QE65000 (Ocean Optics, Orlando, FL, USA) with a wavelength resolution of 1.6 nm and objective 100 × (NA = 0.8). The experimental data are presented as the reflection ratio R_str_/R_ref_, where R_str_ is the reflection spectra obtained from the structures with films, and R_ref_ is the reflection reference from a silver mirror. The white light image (1600 × 1200 pixels) was captured with a digital color camera.

### 2.4. Ellipsometry

The dielectric functions of the 3–9 nm thick Au films were determined by a spectroscopic ellipsometer (WVASE^®^, J. A. Woollam Co., Lincoln, NE, USA) operating in the wavelength range of 280–1700 nm. The data were collected at multiple incidence angles (65°–75° and a step of 5°). The effective optical constants of the Au films were obtained by analysis of the ellipsometry spectra with 10 nm steps [34]. The optical constants of the graphene [35] were used in the ellipsometry model.

### 2.5. Numerical Calculations of the Reflection and Absorption Spectra

For the numerical analysis of multilayer samples, we employed the transfer-matrix approach for planar structures. The top Au film was modeled as a uniform effective medium for which its dielectric function was calculated using the Maxwell–Garnett approximation:

(1) $$\frac{\varepsilon_{eff}-1}{\varepsilon_{eff}+2}=f\frac{\varepsilon_{Au}-1}{\varepsilon_{Au}+2}$$

where the thickness of the top gold layer was taken from AFM measurements (Table S1). Respectively *f* is the volumetric occupancy of gold in the nanolayer (given by Tables S2–S4), and $\varepsilon_{Au}$ is the dielectric function of the thick gold layer [36]. The bottom optically thick gold mirror was considered semi-infinite.

### 2.6. Raman Measurements

The benefit of using hybrid structures for SERS applications was estimated by a confocal scanning Raman microscope Horiba LabRAM HR Evolution (HORIBA Ltd., Kyoto, Japan) equipped with a linearly polarized laser 632.8 nm, 600 lines/mm diffraction grating, and ×100 objective (N.A. = 0.90). Raman spectra were recorded with 0.26 mW incident powers, spot size of ~0.43 μm, and with an integration time of 3 s at each point. Unpolarized detection was used to provide a significant signal-to-noise ratio. Raman spectra of the dye Crystal Violet (CV) were used as a probe for SERS activity. The concentration of an aqueous 10^−6^ M solution CV was used for covering all samples

## 3. Results

**Figure 1:**
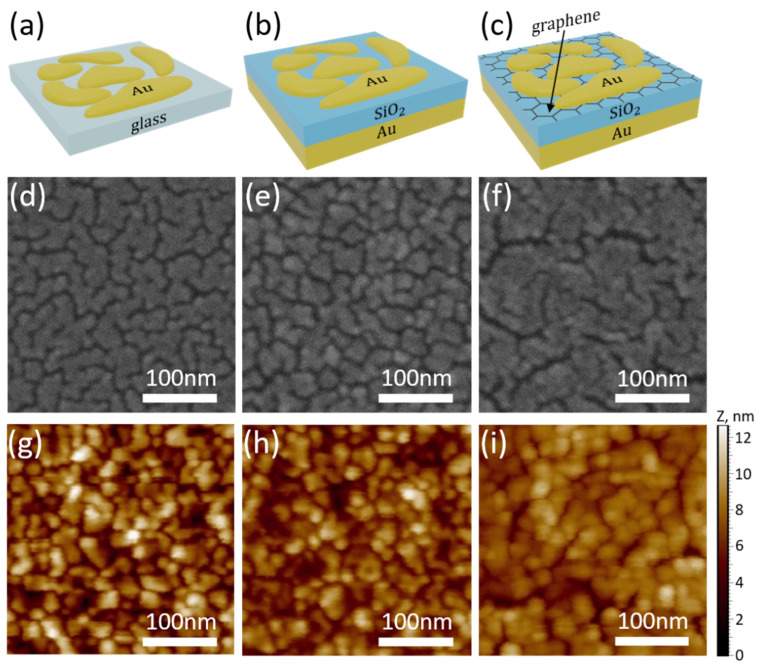
Schematic 3D representation of (**a**) glass/Au, (**b**) film-Au/SiO_2_/Au, and (**c**) film-Au/SiO_2_/graphene/Au substrates, along with (**d**–**f**) SEM images and (**g**–**i**) AFM images of the ultrathin gold 6 nm thick films onto three different substrates. SEM and AFM images for other thicknesses of ultrathin gold can be found in Supplementary materials (Figure S2).

In this study, we provide a systematic study of three types of SERS substrate structures, schematically shown in Figure 1. The first type of structure (Figure 1a), further recalled as glass/Au, is a glass substrate with ultrathin gold films on top of it, which is a classical geometry for SERS structures that has already been well studied [9,15], and the two other types of substrates are compared to it. The second type (Figure 1b), further recalled as film-Au/SiO_2_/Au, is a hybrid structure, and the third type of substrates (Figure 1c), further recalled as film-Au/SiO_2_/graphene/Au, has the same geometry of the second one but distinctly has a transferred single-layer CVD graphene between the dielectric SiO_2_ layer and the upper gold film.

The transferred CVD graphene is mainly a monolayer (more than 95%) with bilayer islands [37]. The presence of monolayer graphene and quality [38,39] was verified with Raman spectroscopy (Figure S1). The 2D/G Raman band ratio of ~0.5 of the substrates (before deposition of the final gold film) indicates that the sublayer of graphene is indeed a single layer [39,40], and the D/G Raman band ratio of ~0.06 indicates a low concentration of defects [40,41,42]. The optical images (Figure S1 insert) of the substrate with graphene show surface homogeneity. For all three types of structures, the thickness of the top deposited thin Au films was from 3 to 9 nm with a step of 1 nm (see Material and Methods). Note that for the thin Au films near the percolation threshold, the “thickness” refers to the average nominal effective thickness measured by the quartz oscillator installed in an evaporation system.

The roughness and actual thicknesses of the deposited gold films were measured by AFM, and the obtained results were compared with the quartz sensor. The date of the AFM measurements of the gold films on glass substrates is presented in Table S1. The growth dynamics of thin films on various surfaces, including graphene, are described in detail enough [43,44,45]; therefore, we only mention the main points. The speed of the growth of initially formed individual Au clusters is faster along the substrate than along the height. Thus, they gradually start to form a labyrinthine structure with thickness increases and eventually form an almost continuous film (Figure S2). The dependence of the cluster size and surface coverage depending on the thickness of the deposited film is presented for all three types of substrates in Tables S2–S4. It is important to note that the morphology of ultrathin gold film is maintained similarly for the glass and film-Au/SiO_2_ substrates, which is critical for comparing the structures of glass/Au and sandwiches (Figure 1d,e,g,h). The morphology of ultrathin gold film (Figure 1f,i) for the film-Au/SiO2/graphene/Au structure looks a little bit different due to the fact the presence of the graphene layer can affect the growth dynamics of thin films and the thickness of the percolation threshold [15,43].

**Figure 2:**
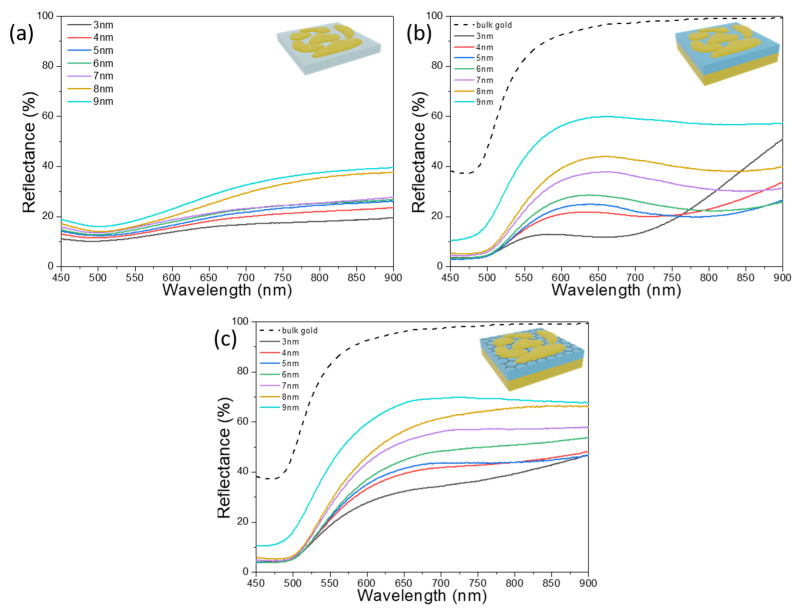
Reflection spectra of the fabricated structures with different thicknesses of gold films for (**a**) glass/Au, (**b**) Au/SiO_2_/Au, and (**c**) Au/SiO_2_/graphene/Au. All of the achieved spectra were normalized to the reflection spectrum of a silver mirror.

The optical properties of the manufactured structures were characterized by reflection spectroscopy (Figure 2) prior to covering the samples with Crystal Violet dye. As expected intuitively, for the glass substrate, film reflectivity increases gradually and monotonously with increasing gold film thickness and without observing any specific resonances (Figure 2a). The reflection spectra resemble bulk gold (the dotted curve in Figure 2b,c), and the spectral features become more expressed with the increase in Au film thicknesses. In the case of hybrid structures, the situation is completely different. The gold film is deposited on the SiO_2_ layer with the reflector—the bottom layer of bulk gold (Figure 1b,c). Despite the presence of a reflector, strong absorption is observed along with the presence of resonances, which are more pronounced for a film thickness of 3 nm and can shift to the red region as the nominal thickness of the Au film increases (Figure 2b). The resonant features of similar structures associated with the percolated film are attributed to the occurrence of localized SP resonances of isolated gold clusters and were described in detail [9,14,15].

In general, for hybrid structures such as film-Au/SiO_2_/Au (Figure 1b,c), several different physical processes contribute to all spectral characteristics of the system. For the long wavelengths (over 750 nm), absorption is mainly provided by the upper gold film. On intermediate wavelengths, between 500 nm and 750 nm, there are three mechanisms, i.e., direct absorption of the top nano-Au, SP modes propagating along with SiO_2_-Au interfaces, and relatively weakly localized gap-surface plasmons (GSPs) between the gold clusters layer and thick bottom layer together, that produce high absorption and the resonant features observed in the spectra [29,46,47,48]. A qualitative insight on the role of these mechanisms can be obtained from the transfer-matrix calculations of absorbance and reflectance (Figure S3). During calculations, we treated the top nano-Au as a uniform effective layer (see Methods for more details). Our simulations agree well with experimental results (Figure 2b) below 550 nm. Upon a further increase in wavelength, the experimentally measured reflectance deviates from the rising trend predicted by simulations since the numerical calculations ignore the complex morphology of the top Au film near the percolation threshold. As a result, resonant absorption on gold clusters in the top nano-Au is predicted at 550 nm (Figure S3), while the reflectance dip is red-shifted to 630–850 nm in the experiment depending on the thickness of the nano-Au film. The red-shift of local SP resonance is explained by the complex non-spherical shape of gold clusters. Additional unaccounted broadband absorption is due to the excitation of GSPs by nano-Au corrugations.

**Figure 3:**
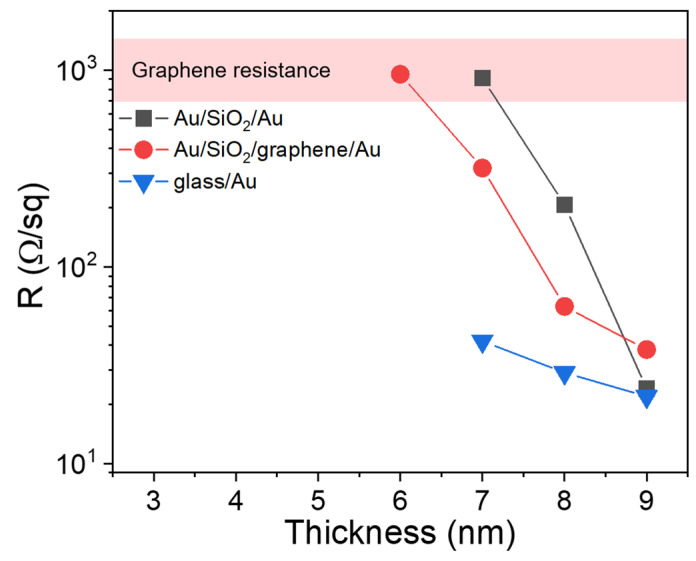
The sheet resistance (R) of Au films as a function of their thickness measured for the three types of structures.

The percolation threshold for gold films on a glass substrate can be determined by measuring sheet resistance [14,44], and for this reason electrical measurements were performed by a four-probe method. The average surface resistance values for each thickness of the Au film are presented in Figure 3. It demonstrates that Au films deposited on glass substrates started conducting at a thickness of 6 nm. Therefore, the percolation threshold for these films lies between 6 and 7 nm. The determination of the percolation threshold for substrates with graphene is more difficult due to the very high electrical conductivity of graphene, which results in conducting samples with graphene at all gold film thicknesses. For Au/SiO_2_/Au hybrid substrates, conductivity is also observed at all thicknesses, which complicates the determination of the percolation threshold of gold films on this type of substrates as well. The noted difficulties are associated with the fact that the probe needles are likely to push through a thin layer of SiO_2_ to the lower continuous layer of gold. The sheet conductivity of graphene was measured separately, and its value varied from 600 to 1700 Ω/square. For hybrid structures with graphene, the deposited Au films are considered conductive when total sheet resistance is less than the minimum sheet resistance of graphene. In accordance with this criterion, the percolation threshold can be estimated to be between 5 and 6 nm for the Au/SiO_2_/graphene/Au structure and between 6 and 7 nm for glass/Au and Au/SiO_2_/Au structure. Most of the time, electrical measurements are sufficient for unambiguously defining film thickness at which the percolation threshold occurs, but in our case, for a more specific determination of the percolation threshold on hybrid structures, additional techniques are required.

Since we cannot unambiguously determine the thicknesses of the percolation threshold for gold films on some types of substrates only using electrical measurements, ellipsometry was used as an additional method. Ellipsometry measurements provide the effective optical constants of Au ultrathin films, which can be used for the determination of the percolation threshold. As a result of the point-by-point fitting of our model to the experimental ellipsometry data, we obtained the spectra of the effective refractive index *n* and extinction coefficient *k* of gold films. In order to obtain the effective dielectric functions from *n* and *k*, we use the following equations.

(2) Re(ε)=n2−k2

(3) Im(ε)=2nk

The dielectric function spectra of the gold thin films [49,50] were evaluated from data recorded by a variable-angle spectroscopic ellipsometer (see Material and Methods). The real part of the dielectric function of the Au film becomes negative in the entire measured wavelength range for films starting from 8 nm for the Au/SiO_2_/Au substrates (Figure S4a), from 6 nm for the Au/SiO_2_/graphene/Au substrate (Figure S4b), and 6 nm for the glass/Au substrate (Figure S4c). This behavior of the curves is more consistent with continuous films [49]. By analyzing the behavior of the graphs characterizing the imaginary part of the dielectric function, we can observe that with an increase in the thickness of the Au film, all graphs have different shapes, and transient processes occur; however, by starting from a distinct thickness, the curves have a similar shape, and the transient processes stop. For Au films on a glass substrate, the graphs show more typical behavior for continuous films starting from 6 nm, for films on Au/SiO_2_ substrates after 8 nm, and for Au/SiO_2_/graphene substrates after 6 nm.

The plasmonic figures of merit (FOM), defined as −Re(ε)/Im(ε), were calculated for the comparison of the metallic optical properties of the deposited Au films.

The FOM curves in the graph grow higher with an increase in film thickness, and their particular behavior well demonstrates the difference between the quality [51] of glass/Au films (Figure S5a), Au/SiO_2_/Au (Figure S5b) and Au/SiO_2_/graphene/Au (Figure S5c) in infrared regions for thicknesses from 3 to 9 nm. The slope of these curves in the infrared region changes sign from negative to positive starting at 8 nm for Au/SiO_2_/Au, at 6 nm for Au/SiO_2_/graphene/Au, and at 6 nm for glass/Au. Based on this criterion [14], it can be concluded that the percolation threshold for gold films on a glass substrate is between 6 and 7 nm, which has a good correlation with electrical measurements.

Due to the fact that percolation threshold thickness values obtained from ellipsometry and electrical measurements appear to be the same for gold films on a glass substrate, we can apply the same approach for other types of substrates as well. Therefore, taking the abovementioned results into consideration, it is possible to conclude that the percolation threshold for Au films is between 7 and 8 nm for the Au/SiO_2_/Au structure and between 6 and 7 nm for Au/SiO_2_/graphene/Au structure.

**Figure 4:**
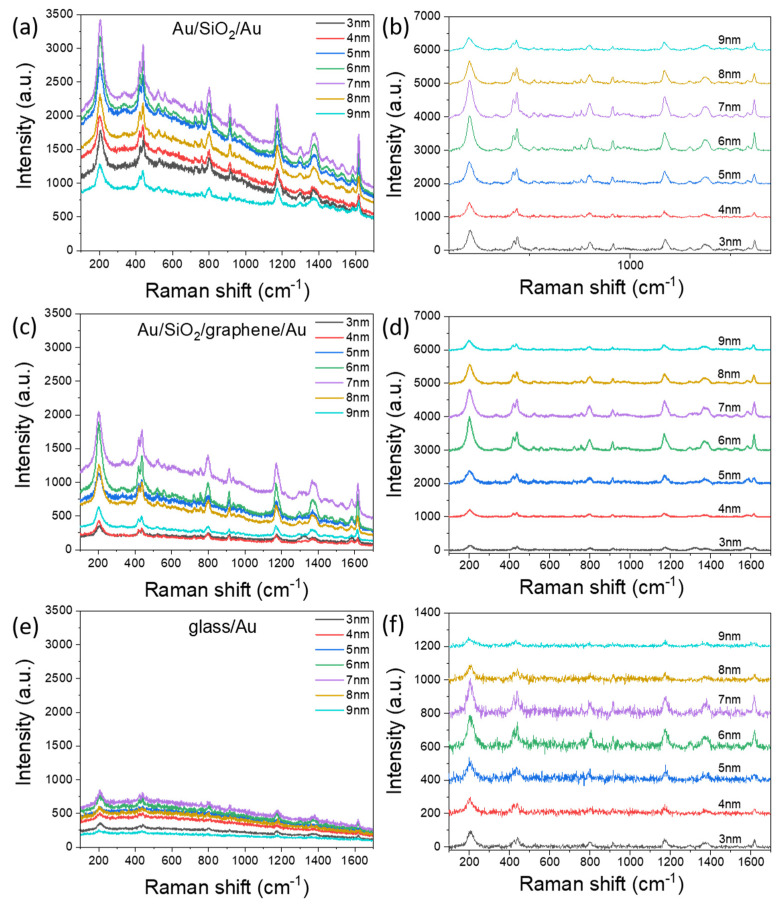
SERS spectra of the Crystal Violet dye with a concentration of 10^−6^ M acquired with a laser excitation of 633 nm. Original spectra and spectra after baseline subtraction for Au/SiO_2_/Au (**a**,**b**), Au/SiO_2_/graphene/Au (**c**,**d**), and glass/Au (**e**,**f**) are both provided.

**Figure 5:**
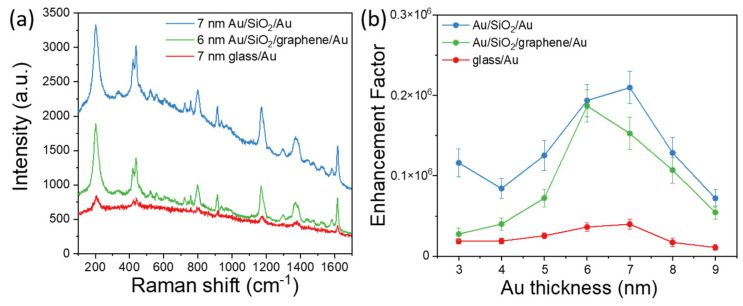
(**a**) SERS spectra of Crystal violet dye obtained for the three types of substrates. (**b**) Obtained SERS enhancement factors as a function of the thickness of the Au film for all three types of substrates, calculated by the intensity of 207 cm^−1^ Raman mode.

After all measurements described above were carried out, Raman microscopy was performed to characterize FE effects in fabricated structures (see Materials and Methods). The used CV is a resonant dye for the 633 nm laser; therefore, it can be a good marker for the estimation of luminescence suppression by graphene. Typical SERS spectra for all three types of substrates are shown in Figure 4. Raman spectra clearly demonstrated that the intensity of Raman peaks increases when using sandwich configurations in comparison with the semi-continuous metal films on the glass substrate (Figure 4a,c and Figure 5a). As noted above, the observed amplification for sandwiches is associated with the excitation of gap plasmon modes and local SP resonances. Moreover, the back reflection from the bottom Au layer gives rise to a stronger absorbance and, therefore, FE since the excitation beam passes through the top film twice. Moreover, due to interference effects, the FE effects are stronger for the structure with 40 nm thick SiO_2_ (Figure 4a) than for the structure with 20 nm thick SiO_2_ (Figure 4c). This observation agrees with numerical simulations (Figure S3) that predict the strongest absorption in the top gold layer for the structure with a 40 nm thick SiO_2_ layer and the weakest absorption for the Au film on the transparent substrate.

At the same period of time, the SERS spectra of all three types of substrates demonstrate similar features associated with a changing intensity on film thickness. The intensity of the Raman signal gradually increases with an increase in gold film thickness from 3 nm to 7 nm for Au/SiO_2_/Au (Figure 4a,b) and glass/Au (Figure 4e,f) substrates and from 3 to 6 nm for Au/SiO_2_/graphene/Au (Figure 4c,d) substrates. An increase in SERS signal intensity for these films is a result of a local electric field enhancement due to a decrease in the gap between clusters and an increasing number of “hot spots.” The maximum SERS signal was obtained for 7 nm thickness of the gold film on Au/SiO_2_/Au substrate and for 6 nm on Au/SiO_2_/graphene/Au and glass/Au substrates. These thicknesses correspond to the thicknesses determined as the percolation threshold by electrical and optical measurements. The percolation threshold corresponds to island morphology forming “hot spots” in nanogaps between clusters and, consequently, resulting in the maximization of SERS signal intensity. After the percolation threshold, SERS intensity begins to decrease with further increases in film thickness for all types of substrates. However, the SERS signal is still observed due to gold film corrugation, as well as individual clusters that have not yet merged together.

These results correlate well with those previously obtained on similar films [9,14,15]. Although the trend for signal intensity versus film thickness appears similar for all three types of substrates. Let us pay attention to one particular feature: The intensity of the CV spectrum is significantly higher for a 3 nm film in the case of gold-dielectric-gold than for 4 nm film and is comparable to the intensity for a 5 nm film (Figure 4b). The observed difference in the signal agrees with the results of optical resonances. Thus, the observed broad resonance at 675 nm for film 3 nm in the provided reflection spectra is near the 633 nm excitation wavelength (Figure 2b). With an increase in the size of the clusters and the filling density of the surface (Tables S2–S4), as the film thickness approaches the percolation threshold, the resonance shifts to the red region (Figure 2b) far from the excitation wavelength.

Therefore, for the following film’s thicknesses, there is a general tendency of signal amplification (to the thickness of the percolation threshold) without some specific features as in the case of film 3 nm. As is known, Raman scattering and luminescence are two opposing phenomena, and the presence of background signals is the result of laser-induced luminescence. In the case of using SERS substrate, the intensity of luminescence can also be enhanced. With the increase in SERS signal, the luminescence background should also increase, and that is observed for our structures. The luminescence background is stronger for sandwich configurations than for the film on glass structures and has a trend that is comparable to that observed for SERS intensity. Thus, it is important to enhance the SERS signal and suppress the increase in luminescence. The difference in the luminescence intensity for hybrid structures “with” and “without” graphene is well observed from the Raman spectra (Figure 4a,c). In the presence of graphene on the structures, the luminescent background in the SERS spectra from structures is significantly lower (at least ~40%) in comparison with structures without graphene. The reason for the suppression of fluorescence in presence of graphene can be due to the transfer of energy and charges through graphene. The transfer of charges from organic molecules to graphene occurs faster in comparison with the transfer of charges from organic molecules to metals [52,53]. This effect occurs when the molecule of CV is in the contact with the graphene. Thus, the presence of graphene for the sandwich structure makes it possible to obtain a benefit with both an increase in SERS signal and a decrease in luminescence. The above comparison for the intensities of the SERS spectra for films on glass and sandwiches includes the contribution of background luminescence. In order to better understand the advantages of using sandwich-based configurations over films on glass, we carefully subtracted the mean background fluorescence (Figure 4b,d,f).

In order to calculate the SERS enhancement factor (EF), an expression for the analytical gain was used. It quantifies the relation between a signal expected from SERS compared with Raman scattering with the same experimental parameters [54]. The average EF is defined by comparing the signals obtained from the CV dye at a concentration of $C_{RS}$ = 10^−2^ M on a simple glass substrate and the signals obtained with a concentration of $C_{SERS}$ = 10^−6^ M on the substrates with 3 to 9 nm gold films. Enhancement factors were calculated by using the following equation.

(4) $$EF=\frac{I_{SERS}}{I_{RS}}\frac{C_{RS}}{C_{SERS}}$$

Here, $I_{SERS}$ and $I_{RS}$ are the intensities of the peaks in spectra after subtracting the baseline for SERS and normal Raman, respectively. The calculation was performed for the seven main CV Raman peaks (Figure 5b). The maximum value of EF was for Au films near the percolation threshold. The EF for these thicknesses of Au films was calculated as 0.23 × 10^6^ for a 7 nm gold film on Au/SiO_2_/Au substrate, 0.18 × 10^6^ for a 6 nm gold film on Au/SiO_2_/graphene/Au substrate, and 0.39 × 10^5^ for a 6 nm Au film on glass/Au substrate. A possible explanation of the observed increase in EF for a 3 nm Au compared to 4 and 5 nm Au-dielectric-Au films has already been described above.

Thus, the intensity of SERS signal for the best performing hybrid substrates, compared to glass/Au substrate, is on average over ~6 times stronger for the hybrid substrate with graphene and over ~7 times stronger for the hybrid substrate without graphene. The ability of graphene to suppress luminescence also provides a great advantage over the other substrates. Note, that SERS performance of our structure might be further optimized by the variation of dielectric thickness (Figures S6–S8) or the size and roughness of the clusters (which can be obtained by varying the deposition parameters of the films and post-fabrication annealing). Another method for optimization is the use, for instance, of nanoporous metals that have been recently introduced [55]. This optimization is a promising direction for further experimental and theoretical investigation.

## 4. Conclusions

Here, we demonstrated a novel approach to the amplification of SERS signal based on the synthesis of hybrid plasmonic structures. This investigation is combined with a detailed investigation of structural and optical properties of gold films near the percolation threshold, deposited as a topmost part of three types of substrates-glass, Au/SiO_2_, and Au/SiO_2_/graphene. The percolation threshold was defined by two methods as a four-probe sheet resistance measurement and spectroscopic ellipsometry. According to these methods, the thickness of the percolation threshold is 6 and 7 nm for the glass/Au and Au/SiO_2_/Au substrates and 5 and 6 nm for the Au/SiO_2_/graphene/Au structures, respectively.

The maximum SERS enhancement factors were achieved for the Au films near the percolation threshold for all three types of substrates. The hybrid structures where a dielectric layer was sandwiched between a thin gold film and an additional thick gold layer showed EFs higher than simple glass/Au substrates by ~7 times for the hybrid structures without graphene and by ~6 times for the hybrid structures with graphene. The capability of graphene to suppress the luminescence background for the fabricated hybrid structures by an average of ~40% clearly demonstrates the benefit of including graphene in hybrid structures. Thus, the demonstrated results highlight the ability of hybrid SERS platforms among the others for the sensitive detection of a wide class of analytes using higher energy lasers in the visible region instead of IR.

## Data Availability

Not applicable.

## Supplementary Materials

The following are available online at https://www.mdpi.com/article/10.3390/nano11123205/s1, Figure S1: Raman spectra of transferred on substrates CVD graphene; Figure S2: SEM images of gold films with thicknesses from 3 to 9 nm deposited on (**a**) glass, (**b**) Au/SiO2, and (**c**) Au/SiO2/graphene structures; Table S1: AFM measurements of the gold film thickness of glass/Au structures; Table S2: Average parameters of ultrathin gold films on glass/Au structures; Table S3: Average parameters of ultrathin gold films on Au/SiO_2_/Au structures; Table S4: Average parameters of ultrathin gold films on Au/SiO_2_/graphene/Au structures; Figure S3. Calculated spectra of reflectance, and absorbance in different gold layers in the Au/SiO_2_/Au setup with h(sensor) = 3 nm and SiO_2_ layer with a thickness of (**a**) 40 nm and (**b**) 20 nm. The optical properties of the sub-percolation gold layer were homogenized using the Maxwell–Garnett approximation. (**c**) Absorbance and reflectance of an Au/SiO_2_ system without the bottom semi-infinite gold layer; Figure S4: Dependence of the real and imaginary parts of the dielectric function of the thin gold films in (**a**,**d**) Au/SiO_2_/Au, (**b**,**e**) Au/SiO_2_/graphene/Au, and (**c**,**f**) glass/Au structures; Figure S5: Ellipsometric figures of merit (FOM) of the thin gold films in (**a**) Au/SiO_2_/Au, (**b**) Au/SiO_2_/graphene/Au, and (**c**) glass/Au structures; Figure S6. Reflection spectra of the fabricated structures with different thicknesses of gold films for Au/SiO_2_/Au substrates with (**a**) 20 nm and (**b** 40 nm thick SiO_2_ layer. All of the achieved spectra were normalized to the reflection spectrum of a silver mirror; Figure S7: SERS spectra of the Crystal Violet dye with a concentration of 10^−6^ M, with a laser excitation of 633 nm for (**a**,**b**) Au/SiO_2_/Au and (**c**,**d**) Au/SiO_2_/graphene/Au structures with 20 nm and 40 nm SiO_2_ thickness; Figure S8: Dependence of SERS enhancement factors (EF) on the thickness of the Au film for Au/SiO_2_/Au substrates with different SiO_2_ layer thicknesses (20 nm, 40 nm, and 50 nm), calculated by the intensity of 207 cm^−1^ Raman mode.
