# Supplementary material for: Hybrid Metal-Dielectric-Metal Sandwiches for SERS Applications
Source: Nanomaterials (Basel). 2021 Nov 26;11(12):3205. doi: 10.3390/nano11123205 (PMC8708964; doi:10.3390/nano11123205)
Supplement: Supplementary file 1 [file nanomaterials-11-03205-s001.zip › nanomaterials-1463866-supplementary.pdf]

nanomaterials

Article

Hybrid Metal-dielectric-metal sandwiches for SERS applications

Mikhail K. Tatmyshevskiy[1,*], Dmitry I. Yakubovsky[1], Olesya O. Kapitanova[1,2], Valentin R. Solovey[1], Andrey A. Vyshnevyy[1], Georgy A. Ermolaev[1], Yuri A. Klishin[1], Mikhail S. Mironov[1], Artem A. Voronov[1], Aleksey V. Arsenin[1], Valentyn S. Volkov[1] and Sergey M. Novikov[1,*]

[1] Center for Photonics and 2D Materials, Moscow Institute of Physics and Technology (MIPT), 9 Institutsky Lane, Dolgoprudny 141700, Russia; dmitrii.yakubovskii@phystech.edu (D.I.Y.); olesya.kapitanova@gmail.com (O.O.K.); valentinsr@mail.ru (V.R.S.); andrey.vyshnevyy@phystech.edu (A.A.V.); ermolaev-georgy@yandex.ru (G.A.E.); klishin.yuri@mail.ru (Y.A.K.); mironov.ms@phystech.edu (M.S.M.); voronov.artem@gmail.com (A.A.V.); arsenin.av@mipt.ru (A.V.A.); vsv.mipt@gmail.com (V.S.V.)

[2] Department of Chemistry, Lomonosov Moscow State University, 1-3 Leninskiye gory, Moscow 119991, Russia

* Correspondence: mikhail.tatmyshevskiy@phystech.edu (M.K.T.); novikov.s@mipt.ru (S.M.N.); Tel.: +79056137678 (M.K.T.); +79032360487 (S.M.N.)

Citation: Tatmyshevskiy, M.K.; Yakubovsky, D.I.; Kapitanova, O.O.; Solovey, V.R.; Vyshnevyy, A.A.; Ermolaev, G.A.; Klishin, Y.A.; Mironov, M.S.; Voronov, A.A.; Arsenin, A.V.; et al. Hybrid Metal-Dielectric-Metal Sandwiches for SERS Applications. *Nanomaterials* **2021**, *11*, 3205. https://doi.org/10.3390/nano11123205

Academic Editor: Maurizio Muniz-Miranda

Received: date
Accepted: date
Published: date

Publisher's Note: MDPI stays neutral with regard to jurisdictional claims in published maps and institutional affiliations.

Copyright: © 2021 by the authors. Submitted for possible open access publication under the terms and conditions of the Creative Commons Attribution (CC BY) license (https://creativecommons.org/licenses/by/4.0/).

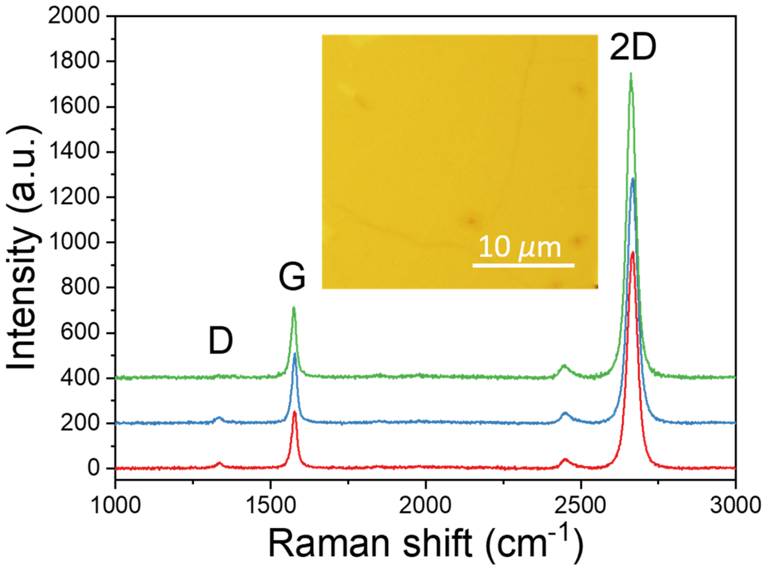

Figure S1. The typical Raman spectra of monolayer CVD graphene on top of an Au/SiO_2 substrate after transfer. The low intensity of D peak demonstrates the high quality of CVD graphene. Insert: the optical image of the substrate with graphene after transferring.

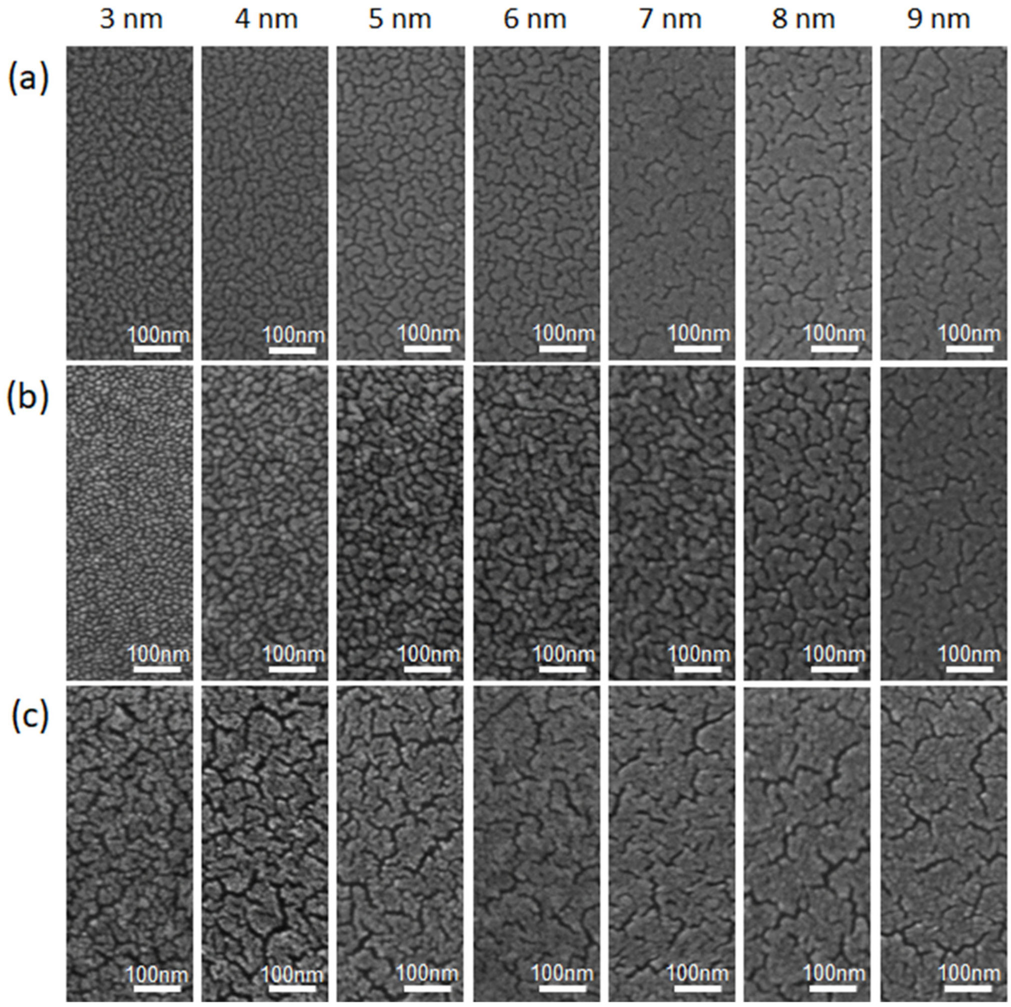

Figure S2. SEM images of gold films with thicknesses from 3 to 9 nm deposited on (**a**) glass/Au, (**b**) Au/SiO_2/Au and (**c**) Au/SiO_2/graphene/Au substrates.

Table S1. AFM measurements of the gold film thickness of glass/Au structures.

h(sensor), nm	glass/Au	
	h, nm	MSE, nm
3	4.3	0.3
4	6.1	0.3
5	6.2	0.3
6	8.7	0.3
7	8.3	0.2
8	9.1	0.3
9	9.7	0.3

Table S2. Average parameters of ultrathin gold films on glass/Au structures.

h(sensor), nm	occupancy, %	average particle size, nm
3	51.1	11.3
4	53.1	15.4
5	58.0	20.0
6	66.3	36.3
7	71.5	—
8	75.4	—

9	81.2	—

Table S3. Average parameters of ultrathin gold films on Au/SiO_2/Au structures.

h(sensor), nm	occupancy, %	average particle size, nm
3	49.3	9.3
4	53.8	17.2
5	56.4	19.6
6	63.7	34.8
7	67.7	—
8	71.3	—
9	79.9	—

Table S4. Average parameters of ultrathin gold films on Au/SiO_2/graphene/Au structures.

h(sensor), nm	occupancy, %	average particle size, nm
3	57.9	28.3
4	64.3	69.2
5	69.0	89.2
6	74.1	—
7	83.1	—
8	85.2	—
9	87.9	—

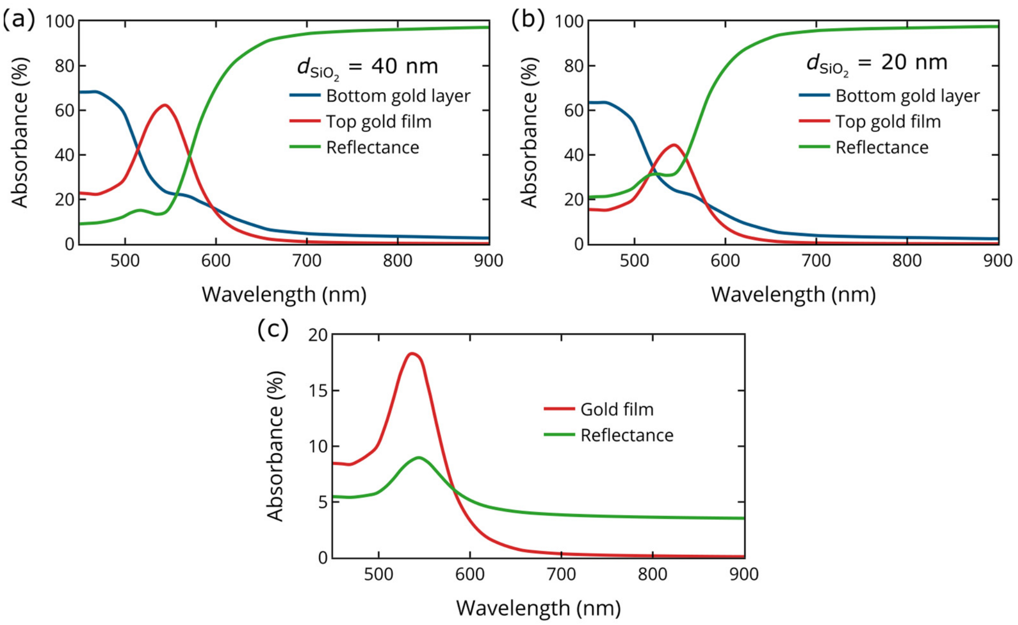

Figure S3. Calculated spectra of reflectance, absorbance in different gold layers in the Au/SiO_2/Au structure with h(sensor) = 3 nm and SiO_2 layer with a thickness of (a) 40 nm and (b) 20 nm. The optical properties of the sub-percolation gold layer were homogenized using the Maxwell-Garnett approximation. (c) Calculated absorbance and reflectance of the nano-Au film on a semi-infinite SiO_2 substrate.

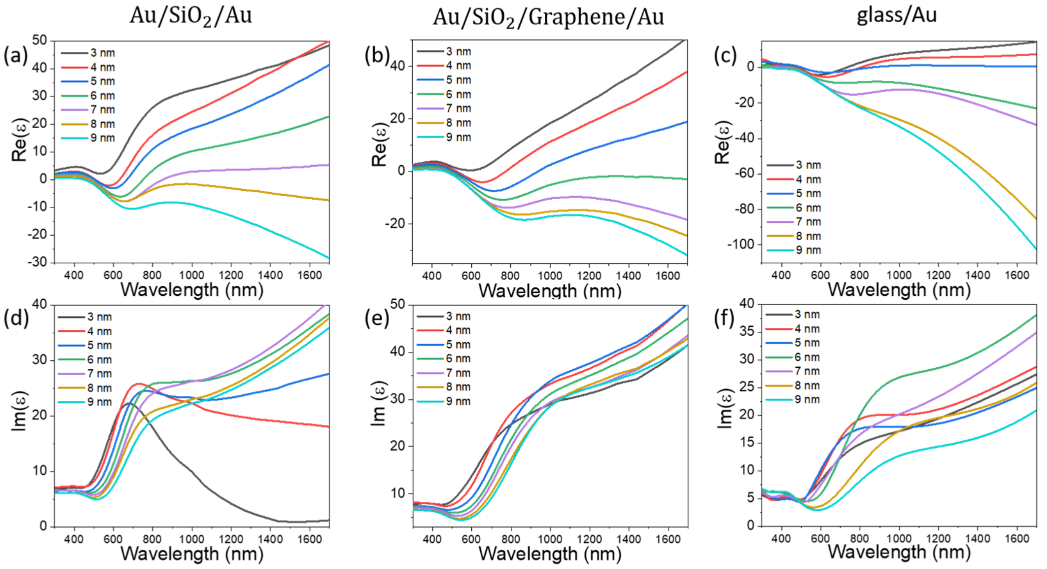

Figure S4. Dependence of the real and imaginary parts of dielectric function of the thin gold films in (a,d) Au/SiO2/Au, (b,e) Au/SiO2/graphene/Au and (c,f) glass/Au structures.

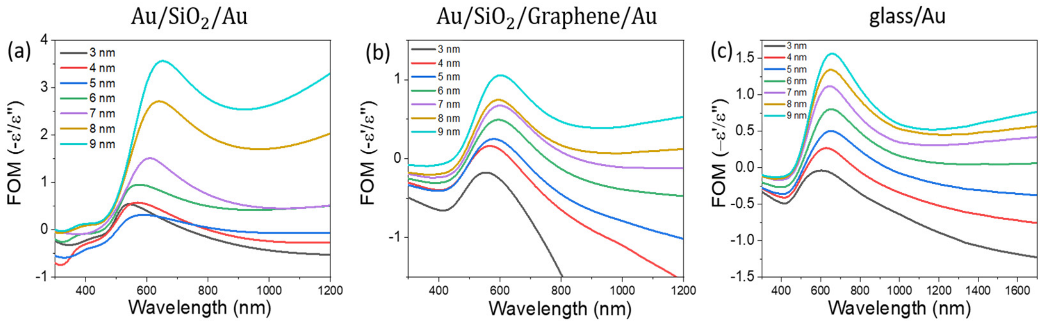

Figure S5. Ellipsometric figures of merit (FOM) of the thin gold films in (a) Au/SiO2/Au, (b) Au/SiO2/graphene/Au and (c) glass/Au structures.

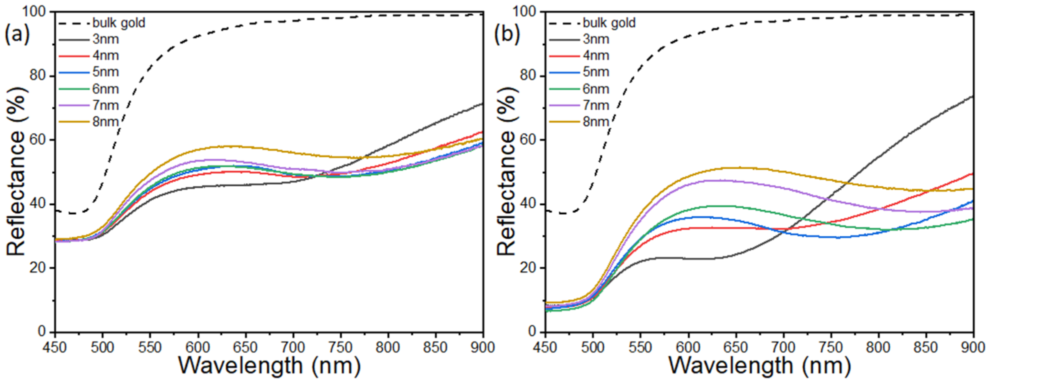

Figure S6. Reflection spectra of the fabricated structures with different thicknesses of gold films for Au/SiO2/Au substrates with (**a**) 20 nm and (**b**) 40 nm thick SiO2 layer. All of the achieved spectra were normalized to the reflection spectrum of a silver mirror.

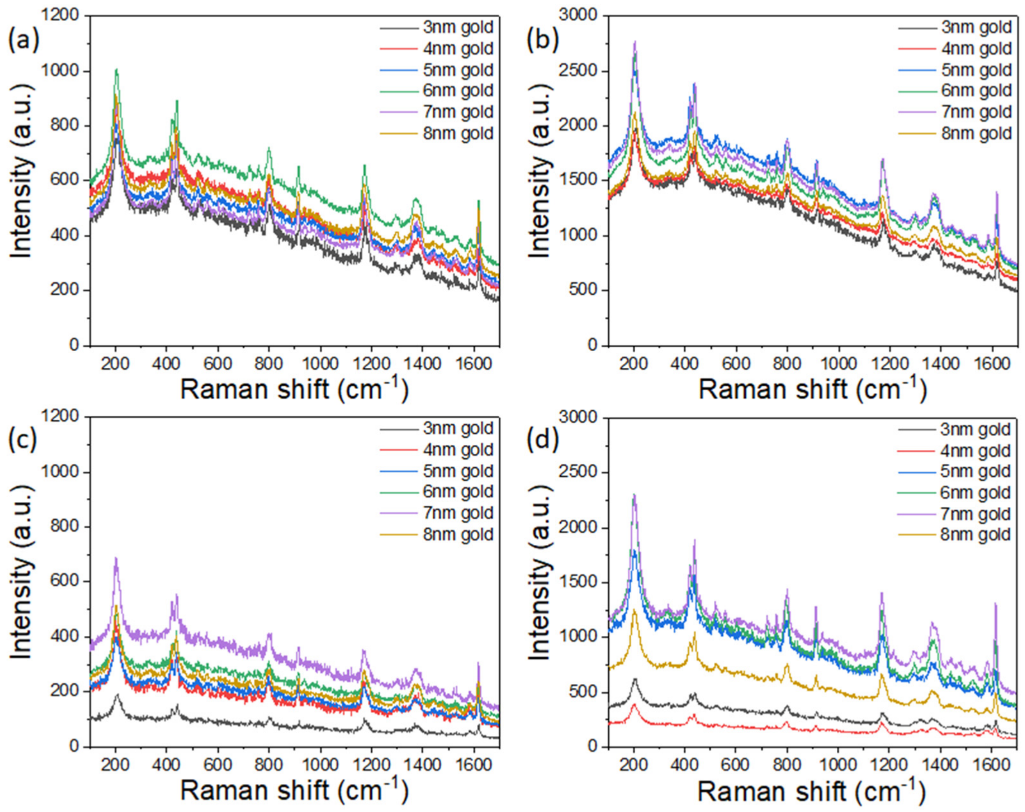

Figure S7. SERS spectra of the Crystal Violet dye with a concentration of 10^{-6} M, acquired with a laser excitation wavelength of 633 nm for (**a**,**b**) Au/SiO_2/Au and (**c**,**d**) Au/SiO_2/graphene/Au structures with 20 nm and 40 nm SiO_2 thickness.

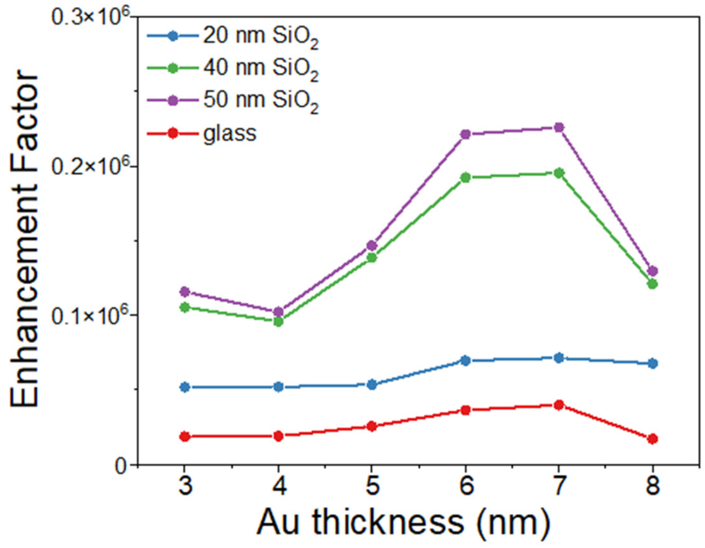

Figure S8. Dependence of SERS enhancement factors (EF) on the thickness of the gold film for Au/SiO_2/Au substrates with different SiO_2 layer thickness (20 nm, 40 nm and 50 nm), calculated by the intensity of 207 cm^{-1} Raman mode.
